# Ideas for Extending the Approach to Evaluating Health in All Policies in South Australia: Comment on "Developing a Framework for a Program Theory-Based Approach to Evaluating Policy Processes and Outcomes: Health in All Policies in South Australia"

**DOI:** 10.15171/ijhpm.2018.25

**Published:** 2018-03-18

**Authors:** Ketan Shankardass, Patricia O’Campo, Carles Muntaner, Ahmed M. Bayoumi, Lauri Kokkinen

**Affiliations:** ^1^Wilfrid Laurier University, Waterloo, ON, Canada.; ^2^Centre for Urban Health Solutions, Li Ka Shing Knowedge Institute, Toronto, ON, Canada.; ^3^Dalla Lana School of Public Health, University of Toronto, Toronto, ON, Canada.; ^4^Bloomberg School of Nursing, University of Toronto, Toronto, ON, Canada.; ^5^Department of Medicine and Institute of Health Policy, Management and Evaluation, University of Toronto, Toronto, ON, Canada.; ^6^Faculty of Social Sciences, University of Tampere, Tampere, Finland.; ^7^Finnish Institute of Occupational Health, Tampere, Finland.

**Keywords:** Health in All Policies, Health Equity, Systems Theory, Developmental Evaluation, Implementation Science

## Abstract

Since 2008, the government of South Australia has been using a Health in All Policies (HiAP) approach to achieve their strategic plan (South Australia Strategic Plan of 2004). In this commentary, we summarize some of the strengths and contributions of the innovative evaluation framework that was developed by an embedded team of academic researchers. To inform how the use of HiAP is evaluated more generally, we also describe several ideas for extending their approach, including: deeper integration of interdisciplinary theory (eg, public health sciences, policy and political sciences) to make use of existing knowledge and ideas about how and why HiAP works; including a focus on implementation outcomes and using developmental evaluation (DE) partnerships to strengthen the use of HiAP over time; use of systems theory to help understand the complexity of social systems and changing contexts involved in using HiAP; integrating economic considerations into HiAP evaluations to better understand the health, social and economic benefits and trade-offs of using HiAP.

Since 2008, the state of South Australia has implemented a Health in All Policies (HiAP) approach under the leadership of the Department of the Premier and Cabinet along with a team of civil servants in the Department of Health (SA Health). Among other activities, a health lens analysis tool has been used to incorporate health planning into the targets of the South Australia Strategic Plan (2004).^1^ For the first five years of HiAP implementation, a research team was partnered with the government for evaluation purposes. In this issue, Lawless and colleagues describe how they used a partnered approach to design a theory-based evaluation of HiAP interventions.^2^

Having a case of HiAP studied in situ is a valuable and rare opportunity for understanding how this upstream health promotion intervention – and its evaluation – works. Although the intervention in South Australia is unique, comprised of an evolving set of governance structures, strategies and tools to implement HiAP, their theory-based approach to evaluation can be used to develop similar bespoke frameworks to evaluate the policy process and outcomes of other HiAPs. Below, we comment on the strengths of the approach described by Lawless and colleagues, and discuss ideas for extending their approach to inform how the use of HiAP is evaluated more generally.

One clear contribution of this study is the use of various models and theories (including institutional theory, agenda setting theory, trust theory, social determinants of health theory, advocacy coalition theory, policy networks and policy learning) from various disciplines (political science, sociology, social epidemiology) to inform their findings. This yields a large number of strategies and mechanisms that explain how HiAP unfolded in South Australia. On the other hand, this framework lacks logical unification. That is, these theories/models represent different (though sometimes not incompatible) epistemologies.^3^ For example, Solar and Irwin’s framework represents a consensus framework in the field of social inequalities in health drafted for a World Health Organization (WHO) Commission^4^; as such it does not include strategies or mechanisms, while social trust theory points to a specific set of social interaction mechanisms. Similarly, Solar and Irwin’s framework bridges various levels while institutional theory and agenda setting remain theory at a macro level. Therefore, the next step in developing the framework adopted by Lawless and colleagues could be theoretical integration between different models to bring about a more broadly coherent understanding of HiAP implementation and outcomes.

The choice of theories used by Lawless and colleagues reflects a disciplinary “standard textbook” or the “received view,” eg,^4-8^. These might be the logical choice to researchers from the different disciplines involved; for example, Kingdon’s multiple stream model is well-read among social epidemiologists and those in public health policy. Future work to understand these strategies and mechanisms unearthed by the authors will benefit from transdisciplinary approaches that integrate other ideas from the policy and political sciences.^9,10^ For example, political economy theories can capture major international trends (eg, globalization and financialization) shaping national and local political contexts and the ability of policy-makers to implement HiAP (eg,^11^). This can facilitate an understanding of tensions between actors and sectors integral to HiAP implementation, including relatively well-resourced sectors, such as a health ministry, more poorly-resourced sectors, such as a housing ministry. Elsewhere, we have described how the implementation of HiAP in Finland has been shaped by the political context over the last thirty years due to changing relationships between the state and the market, between the state and international institutions, and between the state and municipalities.^12^

The authors highlight their distinction between program theory and implementation theory, and their interest in learning about mechanisms of HiAP activities inasmuch as they lead to longer-term outcomes. This is akin to an impact evaluation^13^; yet, the process of implementing HiAP initiatives is itself challenging in terms of technical and political processes that must be managed,^9,10^ so we have argued that a focus on HiAP implementation as an end in itself is needed to better understand how and why HiAP works in the context of unique HiAP approaches (ie, governance structures, strategies and tools) in diverse settings.^14^ Therefore, more methods for process evaluation – perhaps that also integrate program theory – are needed.

The framework presented by Lawless and colleagues shares many elements with traditional logic models, which typically characterize inputs (context, resources), activities, and policy/program impacts depicted in a manner that suggests linear progression from strategies to outcomes. Yet, the process of HiAP implementation involves a social system with cross-sectoral collaborations involving many actors and activities. Social systems are complex (involve a minimum of two persons), have a range of relations of varying intensities (economic, power, cultural/technological relations or structure of the system) sustained by mechanisms (eg, production, domination, research).^15^ Systems theory adds the set of relations between the components of the system (ie, the persons) and those outside the system (ie, the effect of the context on the system); accounting for multiple levels (eg, individuals, organizations, cities, regions), organized hierarchically (eg, atoms, cell, organisms, societies). Systems are characterized by circular and not linear causality as well as myriad types of feedback influences (eg, reinforcing, balancing, counterbalancing, backfiring) that shape successful and unsuccessful policy implementation.

To date, systems theories about HiAP have characterized subsystems of the government that are key in the agenda setting and implementation of HiAP (eg, executive subsystem, intersectoral subsystem and intrasectoral subsystem); key actors that shape the reach and pace of implementation (eg, political elites, expert advisors, civil servants); and other subsystem components which influence HiAP (eg, HiAP mandate, political ideology, prior experience with intersectoral action).^16^ A HiAP system also operates in a context (eg, persons outside the HiAP system that interact with the key actors of the HiAP system, such as industry lobbyists and community organizations). While Lawless and colleagues noted that their framework might not be generalizable, one of the values of systems theories are their generalizability and how they promote the identification of key process and outcome metrics. Other benefits of systems theory approaches are that they are designed to advance collective impact of key actors by enabling policy actors to: see and address root causes and not just their symptoms; avoid unintentional consequences; identify organizations and actors who might be unintentionally working toward conflicting goals; and more accurately see the limits and weaknesses of key policy actors.^17-19^ It is not clear how systems theory is best integrated into the use of logic models for evaluating HiAP; however, a starting point may be to explain expected or unexpected progression in terms of systems concepts like emergence, feedback and non-linearity, and to rely on systems frameworks^16^ for their heuristic value in understanding the causes of changes.

A clear strength of the approach to developing the framework by Lawless and colleagues was the engagement with key stakeholders to capture on the ground knowledge of and experience with HiAP; however, it is unclear how involved key stakeholders were in the evaluation activities. For complex and evolving policies characterized by non-linearity and emergence such as HiAP, it would be wise to design an approach to evaluation that enables data to be used to innovate implementation. Developmental evaluation (DE) – “an evaluation approach that can assist social innovators develop social change initiatives in complex or uncertain environments”^20^– should be considered as a model of collaborating to document success, identify and correct challenges, as well as learn about intended and unintended short and long term outcomes. DE relies on theory as it is often a key source of information about how actions in a complex changing and dynamic environments might bring about outcomes.^21^ DE requires that evaluators be embedded with those who are designing and implementing the programs given the rapidity of the data collection, analysis and application of learnings and the opportunity for co-learning that an integrated evaluation and policy team provides.

Evidence from decades of funding in Canadian and US jurisdictions indicates that greater mortality benefits may be derived from marginal increases in spending in social sectors rather than health sectors.^22^ Similar arguments that favour investing in social determinants of health are frequently made to justify HiAP and other intersectoral investments but economic considerations are rarely integrated into evaluations.^23,24^ Economic considerations did not appear to be a main focus of the evaluation by Lawless and colleagues, although they very usefully describe how austerity budgeting led to HiAP programming. In previous work,^23^ we have demonstrated that HiAP implementations rarely include plans for evaluating costs and outcomes, either within the context of HiAP programs (to maximize technical efficiency) or within the context of public spending more broadly (to address the optimal allocation of resources across sectors). In part, this is because the time horizon for evaluating the impact of some HiAP programs will be too long for effective evaluation; and because some benefits may be hard to attribute, are challenging to measure, or may not be recognized within the timeframe of electoral cycles.^25^ In South Australia, the project to improve healthy eating and increase physical activity may take years to demonstrate health benefits; although the costs of the program are incurred at the time of implementation. Thus, one challenge for a program theory model is to define the appropriate economic theory that will guide evaluation of costs and benefits and facilitate economic evaluations.

## Ethical issues

Not applicable.

## Competing interests

Authors declare that they have no competing interests.

## Authors’ contributions

All authors contributed equally to the writing of this manuscript.

## Authors’ affiliations

^1^Wilfrid Laurier University, Waterloo, ON, Canada. ^2^Centre for Urban Health Solutions, Li Ka Shing Knowedge Institute, Toronto, ON, Canada. ^3^Dalla Lana School of Public Health, University of Toronto, Toronto, ON, Canada. ^4^Bloomberg School of Nursing, University of Toronto, Toronto, ON, Canada. ^5^Department of Medicine and Institute of Health Policy, Management and Evaluation, University of Toronto, Toronto, ON, Canada. ^6^Faculty of Social Sciences, University of Tampere, Tampere, Finland. ^7^Finnish Institute of Occupational Health, Tampere, Finland.
